# Benefits of Combined All-Trans Retinoic Acid and Arsenic Trioxide Treatment of Acute Promyelocytic Leukemia Cells and Further Enhancement by Inhibition of Atypically Expressed Transglutaminase 2

**DOI:** 10.3390/cancers12030648

**Published:** 2020-03-11

**Authors:** Károly Jambrovics, Iván P. Uray, Jeffrey W. Keillor, László Fésüs, Zoltán Balajthy

**Affiliations:** 1 Department of Biochemistry and Molecular Biology, Faculty of Medicine, University of Debrecen, 4032 Debrecen, Hungary; jambrovics.karoly@med.unideb.hu (K.J.); fesus@med.unideb.hu (L.F.); 2 Department of Clinical Oncology, Faculty of Medicine, University of Debrecen, 4032 Debrecen, Hungary; uray.ivan@med.unideb.hu; 3 Department of Chemistry and Biomolecular Sciences, University of Ottawa, Ottawa, ON K1N 6N5, Canada; jkeillor@uottawa.ca; 4 MTA DE Apoptosis, Genomics and Stem Cell Research Group of the Hungarian Academy of Sciences, 4032 Debrecen, Hungary

**Keywords:** acute promyelocytic leukemia, all-trans retinoic acid (ATRA), arsenic trioxide (ATO), ATRA and ATO combination treatment, NB4

## Abstract

Randomized trials in acute promyelocytic leukemia patients have shown that treatment with a combination of all-trans retinoic acid (ATRA) and arsenic trioxide (ATO) is superior in efficacy to monotherapy, with significantly decreased mortality. So far, there are little data available to explain the success of the ATRA and ATO combination treatment in molecular terms. We showed that ATRA- and ATO-treated cells had the same capacity for superoxide production, which was reduced by two-thirds in the combined treatment. Secreted inflammatory biomarkers (monocyte chemoattractant protein-1 [MCP-1], interleukin-1 beta [IL-1β] and tumor necrosis factor-α [TNF-α]) were significantly decreased and were further reduced in a transglutaminase 2 (TG2) expression-dependent manner. The amount of secreted TNF-α in the supernatant of NB4 TG2 knockout cells was close to 50 times lower than in ATRA-treated differentiated wild-type NB4 cells. The irreversible inhibitor of TG2 NC9 not only decreased reactive oxygen species production 28-fold, but decreased the concentration of MCP-1, IL-1β and TNF-α 8-, 15- and 61-fold, respectively in the combined ATRA + ATO-treated wild-type NB4 cell culture. We propose that atypical expression of TG2 leads to the generation of inflammation, which thereby serves as a potential target for the prevention of differentiation syndrome.

## 1. Introduction

Acute promyelocytic leukemia (APL) is characterized by the presence of a chromosomal translocation between the retinoic acid receptor-alpha (RARα) gene on chromosome 17 and the promyelocytic leukemia protein (PML) gene on chromosome 15, resulting in the PML-RARα fusion gene. The PML-RARα oncoprotein acts as an abnormal retinoid receptor, repressing transcription and resulting in the suppression of retinoic acid-induced myeloid differentiation. All-trans retinoic acid (ATRA) both activates the PML-RARα chimera protein and initiates its proteolysis, resulting in the differentiation of APL leukemic cells [1,2,3].

In the 2000s, arsenic trioxide (As_2_O_3_/ATO) was approved by the Food and Drug Administration (US FDA) to treat APL. Clinical data have shown that ATO, either as a single agent or combined with ATRA, can improve the outcomes in newly diagnosed and relapsed APL, compared to ATRA treatment alone [4,5,6].

When combined with ATRA and chemotherapy, ATO can induce complete remission of APL patients, with a five-year overall survival rate up to 90% [5]. At the molecular level, ATO exhibits cytotoxic effects in a concentration-dependent manner, while at lower concentrations (< 0.5 μM), ATO can induce partial differentiation and at higher concentrations (> 0.5 μM), it initiates apoptosis of APL cells. Some of the signal transduction pathways and transactivations of transcription factors involved are arsenic-induced and redox-sensitive. These include the mitogen-activated protein kinases (MAPKs), extracellular signal-regulated kinases 1/2 (ERK1/2), stress-activated protein kinase/Jun-N terminal protein kinases (SAPK/JNK), the apoptosis signal-regulating kinase 1/thioredoxin (ASK1/TRX) system, the AKT-mTOR pathway, transcription factor AP-1 and the nuclear factor kappa-light-chain-enhancer of activated B cells (NF-κB) [7]. Although both ATO and ATRA prime the PML-RARα oncoprotein for proteolysis, only ATO is effective as a monotherapy, through the elimination of residual leukemia-initiating cells. The major limitation to ATO treatment is coagulopathy, which is one of the major causes of early death in APL, driven by procoagulant and fibrinolytic factors [8].

TG2 is a member of the transglutaminase family of enzymes, ubiquitously expressed in various organs. It may be localized intracellularly or extracellularly. In the absence of Ca^2+^, TG2 acts as a G-protein (Ghα), a protein disulfide isomerase or a scaffold protein. In the presence of Ca^2+^, TG2 catalyzes protein crosslinking by its Ca^2+^-dependent transamidation activity [9,10,11,12,13].

Under pathological conditions, elevated TG2 activity is thought to promote various disease states. The TG2 level is markedly increased in tumor cell lines, and its expression is associated with increased metastasis and drug resistance [14,15]. TG2 can drive the epithelial-mesenchymal transition and plays an essential role in the acquisition of cancer stem-cell traits [11,16]. Several studies have been published on the cell death and survival properties of TG2. The pro-apoptotic effects of TG2 are associated with the calcium-dependent protein–protein cross-linking activity at high calcium levels. In its survival-promoting attribution, TG2 most often participates as a protein–protein interaction partner in GTP-bound form [17,18]. In earlier studies, others tested a novel irreversible TG2 inhibitor NC9 to identify agents that can selectively target cancer stem cells in tumors [13,19,20]. Our studies demonstrated that elevated protein expression of TG2 could lead to inflammatory reactions [20,21].

During ATRA induced differentiation of the NB4 acute promyelocytic human leukemia cell lines TG2 is mainly expressed in the cytosol and is partly translocated to the cell nucleus [20,22]. We previously demonstrated the correlation of the expression levels of TG2 and inflammatory cytokines with differentiation syndrome (DS) [20,23].

DS, which develops in approximately 5–25% of patients, is a severe life-threatening condition triggered by the release of inflammatory cytokines, such as tumor necrosis factor α (TNF-α), interleukin-1β (IL-1β) and chemokines like monocyte chemoattractant protein-1 (MCP-1) (CCL2) [24]. These cytokines and chemokines are released from neoplastic promyelocytes as they differentiate in response to both ATRA and/or ATO therapy. Endothelium damage with a capillary leak, edema, hypotension, fever, rash and dyspnea, pulmonary infiltrates, pleural or pericardial effusions and tissue infiltration may ensue [24]. In the last decade, dexamethasone or prednisolone prophylaxis has been used for the prevention of DS during the remission induction phase, but it is ineffective in reducing mortality developing from DS [25].

We also previously demonstrated that shRNA-mediated knock-down or genomic deletion of TG2 in differentiating NB4 cell lines can decrease the levels of chemokines and cytokines, whose production is involved in the development of DS [20,23]. As a diminished expression of TG2 in differentiating APL cells may attenuate the ATRA-induced systemic inflammatory response syndrome in DS, we further investigated the role of TG2 in limiting toxicities arising from DS. NC9 is a TG2-selective irreversible inhibitor that blocks the enzyme active site and locks TG2 in an open conformation, thereby abrogating GTP binding as well. [11,17]. Our results showed that inhibition of TG2 by NC9 in ATRA + ATO combined treatment significantly reduces not just reactive oxygen species (ROS) but also proinflammatory cytokines and chemokine production that significantly increases the severity of DS.

## 2. Results

### 2.1. ATO Alters the Cellular Morphology of Differentiating NB4 Cells

Several clinical studies have reported that the morphology of APL cells changed upon ATRA or ATO treatments. Apoptotic and necrotic cells appeared after ATO-induced cell death, exhibiting a variable size and quality of cytoplasm [26].

**Figure 1:**
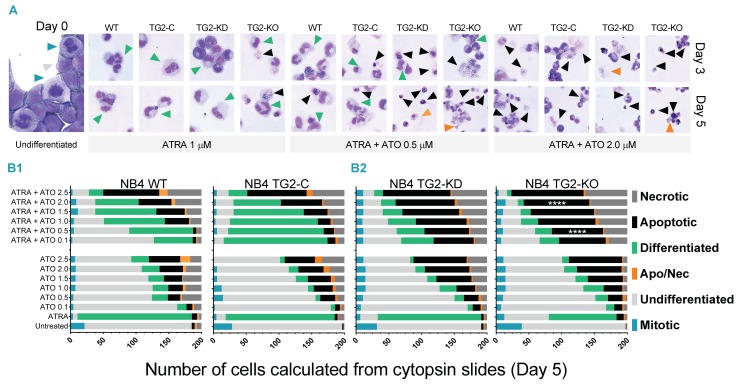
Tissue transglutaminase (TG2) expression moderates arsenic-induced apoptosis. (**A**) TG2 has a protective role against ATO-induced cell death, whereas its absence generates a more sensitive response to the treatment. Representative images of May–Grünwald–Giemsa staining for morphological examination of NB4 cell lines treated with 1 μM ATRA or with ATRA + ATO (0.5 or 2.0 μM) for three and five days (*n* = 5). Light microscopic images and documentation were obtained using the FLoid^®^ Cell Imaging Station instrument (Life Technologies). Cell death features are marked with different triangles based on the color code listed in the lower panel. (**B1**,**B2**) Quantification of May–Grünwald–Giemsa-stained Cytospin™ slides. From each Cytospin slide, 200 cells treated with ATRA or ATO for five days (from 0.1 μM up to 2.5 μM) or in a combination thereof were counted from three different fields of view, were quantified based on cell death features listed and were marked with different colors at the right side of the panels. The graphs represent the mean values of the counted cells, where the black/grey/orange colors mark the cell death features. In NB4 WT and TG2-C cells, ATRA-induced high TG2 levels were associated with lower cell death ratios compared to the TG2-KD or TG2-KO cells. Vehicle controls are in the supplement (Figure S1). Statistical significance was determined via two-way analysis of variance (ANOVA; Bonferroni post-hoc test; ATRA + ATO 2.0 WT: Apoptotic vs. ATRA + ATO 2.0 KO: Apoptotic **** *p* < 0,0001; ATRA + ATO 0.5 WT: Apoptotic vs. ATRA + ATO 0.5 KO: Apoptotic **** *p* < 0.0001).

To determine the differentiation stages of NB4 cells, a variety of morphological changes were evaluated for the single and the combined treatments. In the case of ATRA treatment, the cells mainly represent differentiated and mitotic cells (blue and green triangles, Figure 1A). ATRA, ATO 0.5 μM and the combination treatment caused less apoptosis in NB4 WT and NB4 TG2-C (virus control) cells, such as TG2-deficient NB4 TG2-KD and NB4 TG2-KO cells (Figure 1(B1,B2), upper panels, green and black bars). Results showed that at day 5, arsenic trioxide induces a time- and dose-dependent cytotoxic effect on morphology, representing damaged and late apoptotic-necrotic phases. Higher concentrations of ATO were associated with an increased number of apoptotic and necrotic cells (Figure 1(B1,B2), lower panels, green and black bars). While the number of differentiated cells was low in NB4 WT and NB4 TG2-C cell lines, a higher apoptotic rate was seen in the ATRA + ATO 2.0 μM combined treatment compared to the ATRA + ATO 0.5 μM treatment of cells (Figure 1(B2), upper panels). There was a higher degree of apoptosis or necrosis in NB4 TG2-KD or NB4 TG2-KO cells (Figure 1(B2), upper panel, black and dark grey bars). ATO treatment alone resulted in minor differentiation, with ATO concentration-dependent apoptosis in the NB4 cell lines (Figure 1(B1,B2), lower panels).

### 2.2. ATRA + ATO Combined Treatment Decreases Differentiated NB4 Cells’ Ability to Produce ROS

**Figure 2:**
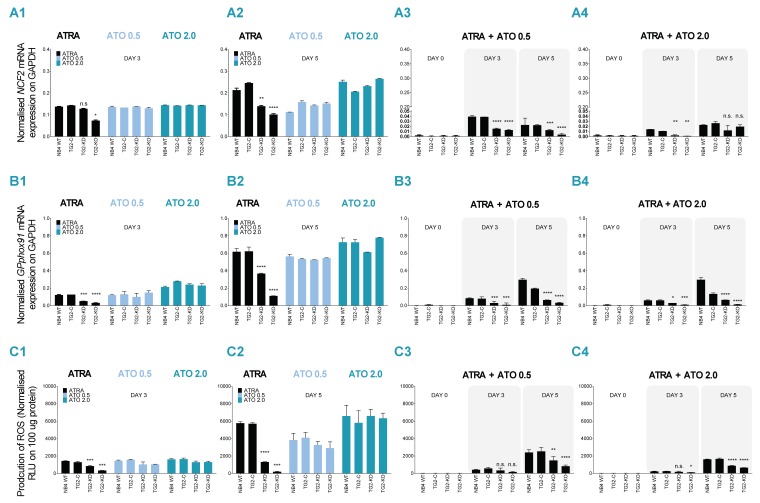
Combined ATRA + ATO treatment attenuates both expression of *NCF2* and *GPPHOX91* respiratory burst oxidase genes and the production of reactive oxygen species. (**A1**–**A4**) NB4 WT, Table. TG2-KD and TG2-KO cells were incubated with 1 μM ATRA, ATO (0.5 μM or 2.0 μM) and a combination of the two (**A3**–**A4**) for three (**A1**) and for five days (**A2**). Relative mRNA expressions of *NCF2/P67PHOX* were measured on the indicated days by real-time Q-PCR and were normalized to *GAPDH*. The graph represents relative mRNA expression (means ± SD, *n* = 3). Statistical significance was determined via two-way analysis of variance (ANOVA; Bonferroni post-hoc test; NB4 WT vs. TG2-KD, TG2-KO * *p* < 0.05, ** *p*< 0.01 and *** *p* < 0.001, **** *p* < 0.0001). **(B1**–**B4**) NB4 WT, TG2-C, TG2-KD and TG2-KO cells were incubated with 1 μM ATRA, ATO (0.5 μM or 2.0 μM) and a combination of the two (**B3**–**B4**) for three (**B1**) and for five days (**B2**). Relative mRNA expressions of *GPPHOX91* were measured on the indicated days by real-time Q-PCR and were normalized to *GAPDH*. The graph represents relative mRNA expression (means ± SD, *n* = 3). Statistical significance was determined via two-way analysis of variance (ANOVA; Bonferroni post-hoc test; NB4 WT vs. TG2-KD, TG2-KO * *p* < 0.05, ** *p* < 0.01 and *** *p* < 0.001, **** *p* < 0.0001). **(C1**–**C4)** NB4 WT, TG2-C, TG2-KD and TG2-KO cells were incubated with 1 μM ATRA, ATO (0.5 μM or 2.0 μM) and a combination of the two **(C3–C4)** for three (**C1**) and for five days (**C2**). Production of ROS was determined for each cell line after the mentioned treatments, using a luminescence-based method in triplicate (*n* = 5), and was reported as RLUs. Graphs are the representation of mean ± SD values normalized to 100 µg protein of cell lysate content. Vehicle controls are in the supplement (Figure S2). Statistical significance was determined via two-way analysis of variance (ANOVA; Bonferroni post-hoc test; NB4 WT vs. TG2-KD, TG2-KO * *p* < 0.05, ** *p* < 0.01 and *** *p* < 0.001, **** *p* < 0.0001).

We previously reported that the atypical expression of TG2 greatly enhances neutrophil granulocytes’ production of ROS by enhancing the expression of two important components of the NADPH-oxidase complex, NCF-2/P67PHOX and GP91PHOX. ATO treatment caused significant cellular changes in NB4 cell lines, which may affect the production of ROS. Because the NADPH-oxidase system is responsible for ROS production, we sought to determine the extent of ROS production after ATRA/ATO treatments. Both NCF-2/P67PHOX and GP91PHOX mRNA expression levels were measured at 1 μM ATRA, 0.5 μM, 2.0 μM ATO, respectively, and ATRA + ATO combined treatments at days 0, 3 and 5. While the levels of mRNS expression of both genes showed a similar pattern, especially on the fifth day, displaying a TG2-dependent expression after ATRA treatment, ATO treatments resulted in a magnitude of gene expression almost similar to that of ATRA generated in NB4 WT cells (Figure 2(A1,A2,B1,B2), left side). In combined treatments (ATRA + ATO, 0.5 and 2.0 μM), as a consequence both of the combined treatment and the extent of TG2 quantities, expression values remained low compared to ATRA or ATO treatments alone (Figure 2(A3,A4,B3,B4), right side). These expression values were also reflected in the production of ROS, especially in the ATRA + ATO 2.0 μM treatment, where a 1/3 ROS generating capacity was measured compared to the ROS production with ATRA or ATO treatment alone, depending on the amount of TG2 (Figure 2(C1–C4)).

### 2.3. Combined ATRA + ATO Treatment Markedly Reduces Inflammatory Biomarker Expression

**Figure 3:**
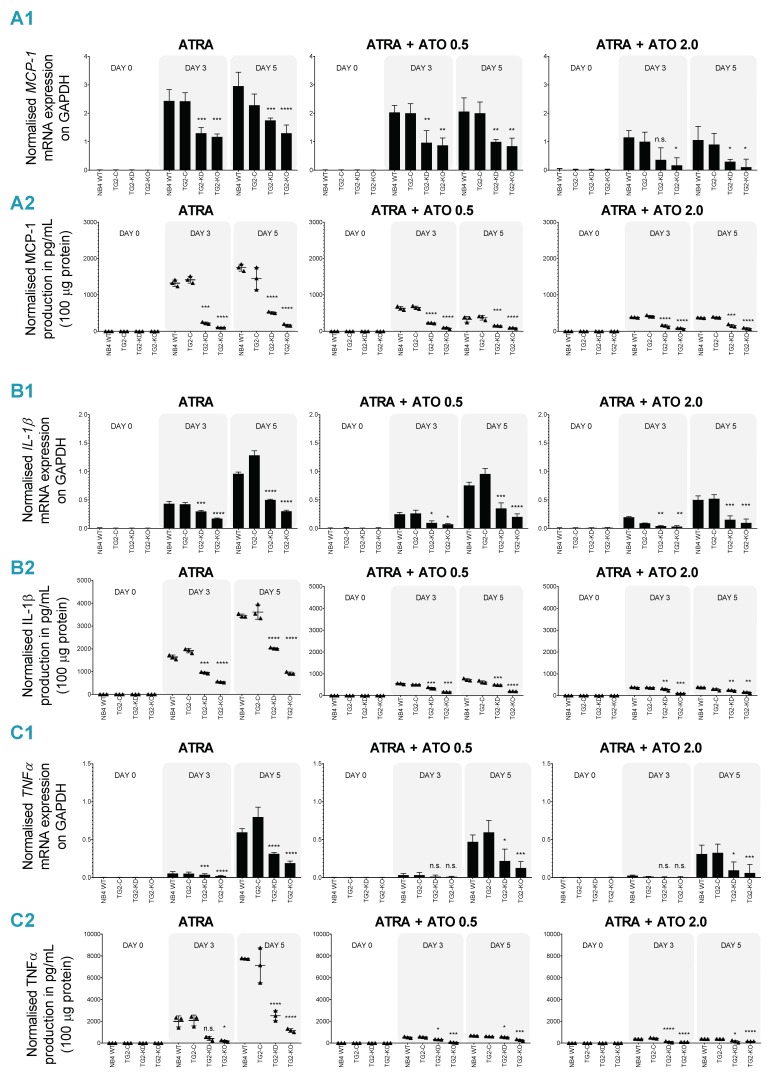
Combined ATRA + ATO treatment can reduce the MCP-1, IL-1β and TNFα inflammatory biomarkers. (**A1,B1,C1**) NB4 WT, TG2-C, TG2-KD and TG2-KO cells were incubated with 1 μM ATRA (left panel), ATO (0.5 μM or 2.0 μM) and a combination of the two (middle and right panels) for three and for five days. Relative mRNA expressions of *MCP-1* (**A1**), *IL-1β* (**B1**) and *TNFα* (**C1**) were measured on the indicated days by real-time Q-PCR and were normalized to *GAPDH*. The graph represents relative mRNA expression (means ± SD, *n* = 3). Statistical significance was determined via two-way analysis of variance (ANOVA; Bonferroni post-hoc test; NB4 WT vs. TG2-KD, TG2-KO * *p* < 0.05, ** *p* < 0.01 and *** *p* < 0.001, **** *p* < 0.0001). (**A2,B2,C2**) The protein content of the supernatants of NB4 cell lines was quantified by ELISA and was normalized to 100 μg protein content of NB4 cell lysates. The secreted protein levels of *MCP-1* (**A2**), *IL-1β* (**B2**) and TNFα (**C2**) from three independent experiments were measured in triplicate and represent the mean ± SD values. Vehicle controls are in the supplement (Figures S3 and S4). Statistical significance was determined via two-way analysis of variance (ANOVA; Bonferroni post-hoc test; NB4 WT vs. TG2-KD, TG2-KO * *p* < 0.05, ** *p* < 0.01 and *** *p* < 0.001, **** *p* < 0.0001).

DS, which can be fatal in 2.5–30% of cases in its moderate or severe forms, is characterized by the presence of a large number of inflammatory, differentiated leukemic cells in the bloodstream that synthesize and secrete chemokines and cytokines, triggering a so-called “cytokine storm.” We previously showed that MCP-1, IL-1β and TNFα were secreted in a TG2-quantity-dependent manner in differentiated NB4 cell lines. MCP-1, IL-1β and TNFα were measured at the mRNA and protein levels in ATRA, ATRA + ATO 0.5 μM, and ATRA + ATO 2.0 μM differentiated NB4 WT, NB4 TG2-C, NB4 TG2-KD and NB4 TG2 KO-cell culture systems. Overall, at day 5, mRNA levels of MCP-1, IL-1β and TNFα were approximately 50% lower for the combined ATRA + ATO 2.0 μM treatment than for ATRA, but these values were further reduced in a TG2-quantity-dependent manner (Figure 3(A1,B1,C1)). At day 5, we observed the effective inhibition of MCP-1, IL-1β and TNFα (with 5×, 10× and 20× lower values, respectively, than the controls) in the case of NB4 WT cells in combined therapy, especially ATRA + ATO 2.0 µM, with respect to proinflammatory cytokines and the chemokine content of cell culture supernatants. These values were further reduced in a TG2-quantity-dependent manner (Figure 3(A2,B2,C2), Table S1).

### 2.4. TG2 Inhibitor NC9 Eradicates the Capability of ATRA + ATO-Differentiated NB4 Cells for ROS and Inflammatory Biomarker Production

**Figure 4:**
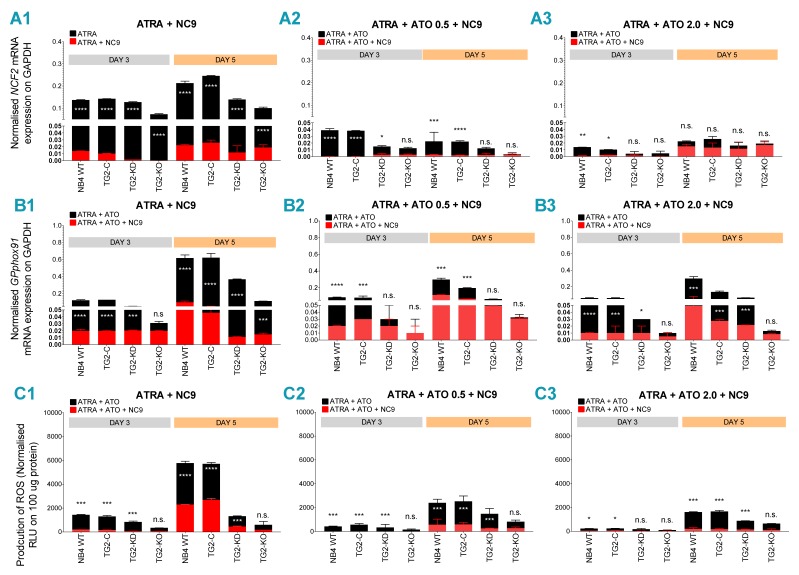
The TG2 inhibitor NC9 diminishes the capability of ATRA + ATO-differentiated NB4 WT cell lines for ROS production. (**A1**–**A3**) Relative mRNA expressions of NCF-2/P67PHOX and GP91PHOX (**B1**–**B3**) were measured in NB4 WT, TG2-C, TG2-KD and TG2-KO cells treated with ATRA + NC9, with ATRA + ATO 0.5 μM + NC9 and with ATRA + ATO 2.0 μM + NC9 at the indicated time points by Q-PCR, and they were normalized to GAPDH (black bars as control, without NC9). The mRNA expression levels from three independent experiments were measured in triplicate, and graphs show the representation of the mean ± SD values. Statistical significance was determined via two-way analysis of variance (ANOVA; Bonferroni and Tukey post-hoc test; ATRA vs. ATRA+NC9 * *p* < 0.05, ** *p* < 0.01 and *** *p* < 0.001, **** *p* < 0.0001). (**C1**–**C3**) In the presence of 30 µM of the TG2 inhibitor NC9, production of ROS was determined for each cell line using a luminescence-based method in triplicate (*n* = 5) and reported in Relative Luminescence Units RLUs without NC9 (black bars as control). Graphs are the representation of mean ± SD values normalized to 100 µg protein of cell lysate content. Statistical significance was determined via two-way analysis of variance (ANOVA; Bonferroni and Tukey post-hoc test; ATRA vs. ATRA+NC9 * *p* < 0.05, ** *p* < 0.01 and *** *p* < 0.001, **** *p* < 0.0001).

NC9 is an irreversible TG2 inhibitor, and ATO-induced differentiation is a TG2-independent process. In cell lines where TG2 was induced by ATRA, significantly lower expression levels of both NCF-2/P67PHOX and GP91PHOX mRNA were measured in the presence of NC9, compared to the controls (Figure 4(A1–A3,B1–B3), red bars, black bars as control). For NCF-2/P67PHOX, the measured values were less than those measured for ATRA in the presence of TG2 inhibitor NC9 (Figure 4(A1–A3)). In ATRA + ATO-treated cells (0.5 and 2.0 μM), TG2 inhibitor NC9 significantly reduced ROS production at day 5 in an ATO concentration-dependent manner (Figure 4(C1–C3)). The ROS production of differentiated NB4 WT cells was decreased 2.4- and 3.5-fold by 0.5 μM and 2.0 μM ATO, respectively. The TG2 inhibitor NC9 further reduced these values to 2.5 times for the ATRA, 10 times for the ATRA + ATO 0.5 µM and 28 times for the ATRA + ATO 2.0 µM treatments (Figure 4(C1–C3)).

**Figure 5:**
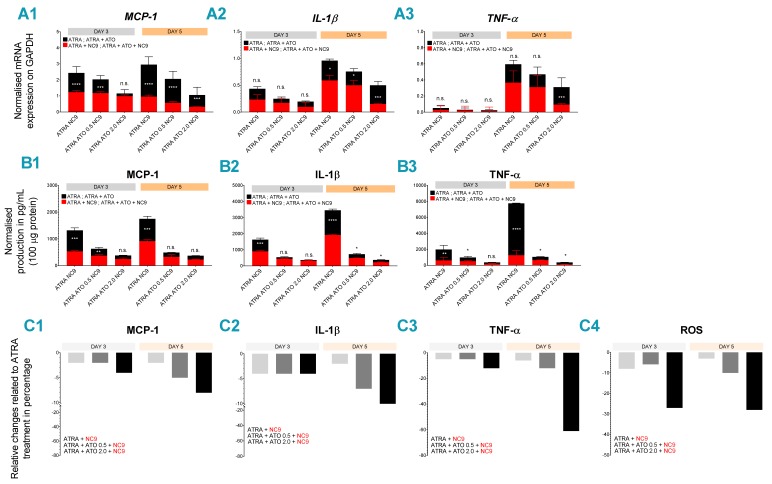
Inhibition of TG2 decreases the gene expression and the protein secretion of TNF-α, IL-1β and MCP-1 in differentiated NB4 cells. Relative mRNA expressions of MCP-1 (**A1**), IL-1β (**A2**) and TNF-α (**A3**) were measured in ATRA and in ATRA + ATO (0.5 or 2.0 μM) in differentiated NB4 cell lines with or without 30 µM NC9 (red bars, black bars as control) on the indicated days by Q-PCR and were normalized to GAPDH. The mRNA expression levels from three independent experiments were measured in triplicate, and the graphs show the representation of the mean ± SD values. Statistical significance was determined via two-way analysis of variance (ANOVA; Bonferroni and Tukey post-hoc test; ATRA vs. ATRA+NC9 * *p* < 0.05, ** *p* < 0.01 and *** *p* < 0.001, **** *p* < 0.0001). The protein content of the supernatants of MCP-1 (**B1**), IL-1β (**B2**) and TNFα (**B3**) with or without 30 µM NC9 (red bars, black bars as control) were quantified by ELISA and were normalized to 100 μg protein content of NB4 WT cell lysates. The figures show secreted protein levels from three independent experiments measured in triplicate, and the graphs show the representation of the mean ± SD values. Statistical significance was determined via two-way analysis of variance (ANOVA; Bonferroni and Tukey post-hoc test; ATRA vs. ATRA+NC9 * *p* < 0.05, ** *p* < 0.01 and *** *p* < 0.001, **** *p* < 0.0001). Relative changes of MCP-1 (**C1**), IL-1β (**C2**), TNFα (**C3**) and ROS (**C4**) production in the presence of NC9 related to ATRA-only treatment in NB4 WT cells. Changes were calculated from values of ATRA only and ATRA + NC9, from ATRA + ATO 0.5 μM + NC9 and from ATRA + ATO 2.0 μM NC9 treatments. Graphs represent the changes compared to a single ATRA treatment.

Since the levels of TG2 expression altered the expression and secretion levels of MCP-1, IL-1β and TNFα inflammatory biomarkers in NB4 WT, NB4 TG2-C, NB4 TG2-KD and NB4 TG2-KO cells after the combined treatment with ATRA + ATO (0.5 and 2.0 μM) (Figure 5(A2,B2,C2)), it seemed reasonable to investigate the effect of TG2 inhibitor NC9 on differentiating NB4 WT cells using the combined ATRA + ATO (0.5 and 2.0 μM) treatment. At day 5, we found that it significantly reduced the quantities of both mRNA and protein of MCP-1, IL-1β and TNF-α in cells and supernatants, especially in the case of TNF-α, compared to results from a single ATRA treatment (Figure 5(A1–A3,B1–B3), red bars, Table S2.)

## 3. Discussion

ATRA-based therapy is frequently used in the clinical treatment of APL patients, which leads to the terminal differentiation of leukemic cells towards the neutrophil granulocyte stage. Hyperinflammatory reactions may constitute severe side effects of ATRA treatment, including the infiltration and damaging of soft tissues and organs, such as the lungs and heart, by differentiating APL cells that produce reactive oxygen species. ATRA-induced differentiation of APL cells could generate increased secretion of cytokines, chemokines and interleukins as well as cell adhesion and migration. ATRA-induced differentiation is also associated with the elevated expression of two components of the NADPH-oxidase complex, NCF-2/P67PHOX and GP91PHOX, resulting in the possibility of increased ROS production and, consequently, more severe organ damage.

It has previously been reported that the morphology of APL cells changed upon ATRA or ATO treatment. ATO treatment was associated with apoptotic or necrotic cell death, exhibiting variable size and quality of cytoplasm [26]. Notably, reduced or absent TG2 enhanced the sensitivity of NB4 cells to a combined ATRA + ATO 2.0 μM treatment, with significantly higher apoptotic and necrotic rates.

Oxidative stress caused by reactive oxygen species, a group of oxygen-based reactive molecules produced by ATO activated the NADPH-oxidase system, resulting in the disruption of mitochondrial membrane potential and subsequent apoptosis [27,28,29]. As published previously, the mRNA expression of NCF2/P67PHOX and GPPHOX91 were substantially higher in the presence of TG2 compared to NB4 TG2-KD and TG2-KO cells. Here we find that ATO alone can trigger an increase in the amount of mRNA of NCF2 and GPPHOX91 as well as the production of ROS, similar to ATRA in differentiated NB4 cell lines. So far, no study has shown a 2-fold decrease in ROS production in response to combined ATRA + ATO compared to single ATRA or ATO treatments. While amounts of atypically expressed TG2 in differentiated NB4 cell lines can enhance the function of the NAPDH-oxidase system, leading to high ROS production, TG2 deficiency in TG2-KD cells or TG2-KO cells is associated with significantly lower ROS production, which may be further reduced by the combined ATO treatments.

Resting neutrophil granulocytes can be activated by cytokines, chemokines, interleukins (inflammatory biomarkers) or integrin-mediated signaling processes. Similarly, differentiating or mature NB4 cells synthesize and secrete cytokines, chemokines or interleukins induced by ATRA treatment [20,23,24,30]. In APL, differentiating APL cells stimulate their own extravasation and migration into various organs by producing inflammatory cytokines, chemokines and interleukins (MCP-1, IL-1β, TNF-α) in an autocrine mechanism.

Compared to ATRA treatment, combining ATRA with ATO reduced both mRNA expression and the concentration of secreted MCP-1, IL-1β and TNF-α in a time-dependent manner (Figure 3(A2,B2,C2)). The decreases directly correlated with both TG2 expression and ATO concentration. The NC9 TG2 inhibitor significantly reduced the mRNA levels of NCF-2/P67PHOX, GP91PHOX, MCP-1, IL-1β and TNF-α and also reduced the production of ROS and secreted levels of MCP-1, IL-1β and TNFα (Figure 4(A1–A3,B1–B3,C1–C3); Figure 5(A1–A3,B1–B3), red bars). The amount of secreted proteins was reduced significantly (to a 7- to 60-fold decrease) after the combined ATRA + ATO treatment compared to the ATRA treatment alone (Table S1).

We have previously shown that the TG2 inhibitor NC9 decreases NF-κB translocation to the nucleus and NF-κB transcriptional activity as well as significantly reducing the production of inflammatory biomarkers, such as MCP-1, IL-1β and TNF-α [20]. Accordingly, our current study demonstrates that combined ATRA + ATO (2.0 µM) with an inhibition of atypically expressed TG2 by NC9 radically limits the expression and secretion concentration of three inflammatory biomarkers and radical oxygen species (Figure 5(C1–C4)). ATRA + ATO (2µM) + NC9 treatment resulted in an 8-fold decrease in MCP-1, a 15-fold decrease in IL-1β levels and a 61-fold suppression of TNFα; there was also a 28-fold reduction in ROS production, compared to ATRA alone, at day 5.

## 4. Materials and Methods

### 4.1. Cell Cultures and Treatments

NB4 cells sublines NB4 TG2-C (virus control containing scrambled shRNA [23], TG2-deficient NB4 TG2-KD (shRNA based knockdown) [23] and NB4 TG2-KO cells (TALEN TG2 knockout) [20] were maintained in RPMI-1640 medium containing 10% fetal bovine serum (Life technologies, Van Allen Way, Carlsbad, CA 92008, United States), 2 mM L-glutamine (Sigma Aldrich, St. Louis, Missouri United States) and 100 μg/mL penicillin-streptomycin solution (Sigma Aldrich). Cells were differentiated in the presence of 1 μM ATRA (Sigma Aldrich). For combined treatments, 1 μM ATRA with either 0.5 μM or 2.0 μM ATO solutions were prepared (Sigma Aldrich). For TG2 inhibitory treatment, NC9 was used at 30 μM final concentration (Stock concentration: 30 mM). Cells were incubated at 37 °C with 5% CO2. Treatments lasted 0–5 days. Other compounds and solutions were used as listed in the Online Table S3.

### 4.2. Cytospin

Samples were taken from a homogeneous suspension of cell cultures. After pre-cleaning the slides (70% alcohol), 10 μL of a homogeneous sample together with 90 μL of 1x phosphate-buffered saline was applied to the Cytospin™ tube (Shandon CYTOSPIN II, 6511 Bunker Lake Blvd. Ramsey, MN 55303 USA), followed by centrifugation at 800 rpm for 3 min. Samples were then fixed at room temperature with methanol and were prepared for further staining.

### 4.3. May-Grünwald Giemsa Staining

May–Grünwald and Giemsa solutions were diluted with distilled water at a 1:10 ratio. Previously fixed samples were stained with a May–Grünwald solution for 10 min and were then rinsed with a diluted Giemsa solution for 5–30 min. Slides were washed and dried at room temperature. Light microscope images and documentation were obtained using a FLoid^®^ Cell Imaging Station instrument (Life Technologies) at a scale of 200 µm. The ratio of the undifferentiated, differentiated, apoptotic, necrotic and mitotic cells in NB4 cell lines was determined by morphological changes/features during the ATRA, ATO and combined ATRA + ATO treatments. Based on our morphological type evaluations, we classified the morphological types of ATRA-, ATO- and ATRA + ATO-differentiated NB4 cells into six groups: a, undifferentiated (unsegmented nuclear region and thin cytoplasmic region); b, differentiated (a segmented nuclear fraction with the white-grey higher proportion of the cytoplasmic region); c, apoptotic (well-defined membrane changes, with shrinkage) and d: necrotic (lack of nuclear fraction with severe destruction of the membrane structure). Other groups, such as: e, apoptotic-necrotic (strong blue nuclear remnants, disarrayed membrane structure) and f, mitotic (chromatin changes, round shape) were also defined from the ATRA and ATO combined treatment.

### 4.4. Gene Expression Analysis

For Q-PCR measurements, total RNA samples were isolated by TRIzol reagent, following the company’s instructions. Total RNA was quantified by a NanoDrop 2000 Spectrophotometer (Thermo Fisher, Waltham, MA USA 02451). Each sample was diluted to 200 ng/μL concentration, followed by reverse transcription using the High Capacity cDNA Reverse Transcription Kit (Thermo Fisher) in a reaction of: 10 μL sample + 10 μL RT-Master mix. The assay and the PCR were performed according to the manufacturer’s protocol. For the real-time Q-PCR reaction, the following TaqMan probes (ABI, Applied Biosystems, Waltham, MA USA 02451) were used: NCF2, GPPhox91, IL-1β, MCP-1, TNF-α and GAPDH. The analysis was carried out using the ABI Prism 7900 (ABI, Applied Biosystems). Relative mRNA expression levels were normalized to the level of GAPDH using the ΔΔCt method.

### 4.5. Superoxide Anion Production

The amount of superoxide radicals was measured by luminol-chemiluminescence assay, using L-012 dye (Wako Pure Chemical Industries, Ltd., 1-2 Doshomachi 3-chome, Chuo-ku, Osaka, Japan) after PMA (50 nM) induction, in a reaction volume of 100 μL medium containing cells and L-012 (50 μM) dye. After 5 min, samples were measured in a Synergy Multimode Microplate Reader (BioTek Instruments, Inc, Winooski, VT, USA). Production of generated light by the reaction was recorded in relative luminescence units (RLUs) and was corrected with the protein concentration levels of the samples.

### 4.6. Statistical Analysis

Statistical analysis was carried out using GraphPad Prism version 8.02, by two-way ANOVA (Bonferroni posthoc test; * *p* < 0.05, ** *p* < 0.01 and *** *p* < 0.001, **** *p* < 0.0001).

## 5. Conclusions

Together, these results suggest that the atypical expression level of TG2 in ATRA-induced differentiating APL cells is a crucial factor in developing inflammation and in the production of ROS. The lower expression of TG2 may effectively reduce the chance of inflammatory processes and organ damage.

In the combined ATRA + ATO treatment of differentiating APL cells, ATO can restrict ATRA-induced ROS and inflammatory biomarker production capacity in a dose-dependent manner. The irreversible inhibitor of TG2 NC9 not only decreased reactive oxygen species production 28-fold, but decreased the concentration of MCP-1, IL-1β and TNF-α 8-, 15- and 61-fold, respectively in the combined ATRA + ATO-treated wild-type NB4 cell culture.

## Supplementary Materials

The following are available online at https://www.mdpi.com/2072-6694/12/3/648/s1, Table S1: Protein content of supernatant of NB4 cell lines, Table S2: Protein content of supernatant of NB4 cell lines, Table S3: List of chemicals and reagents were used, Figure S1: Representative images of May-Grünwald-Giemsa staining, Figure S2: Production of ROS in vehicle treated NB4 cells, Figure S3: mRNA expression of MCP-1, IL-1β and TNFα inflammatory biomarkers, Figure S4: The protein content of the supernatants of NB4 cell lines was quantified by ELISA.
